# Isolation, identification, and pathogenicity analysis of newly emerging gosling astrovirus in South China

**DOI:** 10.3389/fmicb.2023.1112245

**Published:** 2023-02-27

**Authors:** Jingyu Xu, Liguo Gao, Puduo Zhu, Sheng Chen, Zixian Chen, Zhuanqiang Yan, Wencheng Lin, Lijuan Yin, M. Tariq Javed, Zhaoxin Tang, Feng Chen

**Affiliations:** ^1^College of Animal Science, South China Agricultural University, Guangzhou, China; ^2^Guangdong Enterprise Key Laboratory for Animal Health and Environmental Control, Wen's Foodstuff Group Co. Ltd., Yunfu, China; ^3^Faculty of Veterinary Science, University of Agriculture, Faisalabad, Pakistan; ^4^College of Veterinary Medicine, South China Agricultural University, Guangzhou, China

**Keywords:** goose astrovirus, pathogenicity, phylogenetic analysis, goose gout, isolation

## Abstract

Goose astroviruses (GoAstV) cause fatal gout and decrease product performance in the waterfowl industry across the world. Since no effective vaccines are available, studies on the epidemiology of the virus are necessary for vaccine development. In this study, we collected 94 gout samples from goose farms in the Guangdong Province of South China. Among them, 87 samples (92.6%) tested positive for GoAstV, out of which five GoAstV strains were isolated after four generations of blind transmission through healthy 13-day-old goose embryos. The whole genome of the isolates was sequenced and further analyzed by comparing the sequences with published sequences from China and other parts of the world. The results of the alignment analysis showed that nucleotide sequence similarities among the five GoAstV isolates were around 97.4–98.8%, 98.6–100%, 98.1–99.8%, and 96.7–100% for the whole genome, ORF1a, ORF1b, and ORF2, respectively. These results showed that the GoAstV isolates were highly similar to each other, although they were prevalent in five different regions of the Guangdong Province. The results of the phylogenetic analysis showed that the whole genome, along with the ORF1a, ORF1b, and ORF2 genes of the isolates, were clustered on a single branch, along with the recently published GoAstV-2, and were very distinct from the DNA sequences of the GoAstV-1 virus. In this study, we also reproduced the clinical symptoms of natural infection using the GoAstV-GD2101 isolates, confirming that the gout-causing pathogen in goslings was the goose astrovirus. These findings provided new insights into the pathogenicity and genetic evolution of GoAstV and laid the foundation for effectively controlling the disease.

## Introduction

Members of the family Astroviridae are small, non-enveloped, single-stranded RNA viruses. They have a diameter of 28–30 nm and a characteristic five-or-six-pointed star on the surface of about 10% of virions (Madeley and Cosgrove, 1975; Liu et al., 2022). Their genomes are about 6.1 to 7.9 kb long, which include three open reading frames (ORF1a, ORF1b, and ORF2), a 3′-UTR, and a poly A-tail. ORF1a and ORF1b encode non-structural polyproteins, which regulate viral replication. ORF2 encodes the capsid protein, which is responsible for coating viral nucleic acids and nucleic acid-protein complexes and works against viral infections (Cortez et al., 2017; Yang et al., 2018; Li et al., 2022).

The International Committee on Taxonomy of Viruses (ICTV) classified Astroviridae into two viral genera, including *Mamastroviruses* (MAstVs) and *Avastroviruses* (AAstVs; Wohlgemuth et al., 2019; Zhang P. et al., 2022). The mammal astrovirus genus is further divided into six categories, which include human, pig, cat, mink, sheep, and dog astroviruses, and the avian astrovirus genus is divided into three species, including AAstV-1, AAstV-2, and AAstV-3. Among them, AAstV-1 has a single member, which is Turkey astrovirus type 1 (TAstV-1), AAstV-2 includes Nephritis virus (ANV) and the Chicken astrovirus (CAtsv), and AAstV-3 consists of Turkey astrovirus type 2 (TAstV-2), Turkey astrovirus type 3 (TAstV-3), Duck astrovirus type 1 (DAstV-1), Duck astrovirus type 2 (DAstV-2), Duck astrovirus 3 (DAstV-3), Duck astrovirus 4 (DAstV-4), Goose astrovirus 1 (GoAstV-1), and Goose astrovirus 2 (GoAstV-2; De Benedictis et al., 2011). There are many other kinds of avian astroviruses which are not officially classified by the ICTV, such as the avian astrovirus isolated from various wild birds in the tropical rainforest (Fernandez-Correa et al., 2019; He et al., 2020).

A novel disease, characterized by gout, hemorrhage, and swelling of the kidneys affecting goslings, appeared in the Shandong Province of China in 2016. This outbreak was caused by the novel virus GoAstV (Yang et al., 2018). Both GoAstV-1 and GoAstV-2 could cause kidney swelling and visceral gout in goslings (Wang et al., 2021; Zhang F. et al., 2022). The number of cases of GoAstV-infected goslings that developed gout increased in 2017 (Zhang et al., 2018). The morbidity rate of the infected goslings was up to 50%, resulting in high mortality (15–30%). The infection caused the death of thousands of goslings and significant economic loss to communities, owing to the persistence of this condition in certain provinces. Therefore, effective control of GoAstV infections and systematic investigations are necessary to resolve this crisis. An outbreak of a highly lethal disease characterized by gout occurred in major goose breeding areas of Guangdong Province, South China, in the second half of 2021. In this study, we determined the pathogenicity and the phylogenetic relationship between newly emerging GoAstVs, to enhance our understanding of the virus and its prevalence. Our findings provide useful guidelines for effective epidemiological control of GoAstV in China.

## Materials and methods

### Sample collection and goose astroviruses detection

From September 2021 to June 2022, five goose farms located in Qingyuan City, Foshan City, Zhaoqing City, Heyuan City, and Zhanjiang City from South China were sampled, a total of 94 goslings with typical clinical signs were collected. Pathological examination was carried out for gout confirmation. Tissue samples of kidneys, heart, spleens and livers were collected and homogenized to test for the presence of GoAstV, Newcastle disease virus (NDV), Avian orthoreovirus (ARV), Goose polyomavirus (GPOV), Goose circovirus (GoCV), Goose parvovirus (GPV), Avian influenza viruses (AIV-H5, H7, and H9 subtypes) and Duck Tembusu Virus (DTMUV). Detection of GoAstV and other viruses using primers listed in Table 1.

**Table 1:** PCR identification primers for other viruses.

Primers name	Sequence (5′ → 3′)	Annealing temperature (°C)	Product size (bp)
GoAstV-2-F	GAAGAAAAGAGTAGCTGGAC	57	451
GoAstV-2-R	CAAGTGAGTCAGTGGGTGAA		
NDV-F	ATGGGCYCCAGAYCTTCTAC	56	535
NDV-R	CTGCCACTGCTAGTTGTGATAATC		
ARV-F	GCGACGTCGATCATTTGAAG`	55	822
ARV-R	GTGATCGGAAAGAGCCAGTA		
GPOV-F	TGCTCTGTCAGCATTCCATTG	58	1,039
GPOV-R	CTGCTTCACCAGCAAGCACA		
GoCV-F	TGCTGCGCTTGAAGAGAAGC	60	334
GoCV-R	GTAACGGCTCTTCCCGCTTC		
GPV-F	TATCAACAACCATTGGGGAA	56	470
GPV-R	TTCTGCTGCTGTCTACCTCAT		
AIV (H5)-F	GTCAAAATGGAGAAAATAGTG	55	1700
AIV (H5)-F	AACTGAGTGTTCATTTTGTCAAT		
AIV (H7)-F	CCAGCAAAAGCAGGGGATACAAAATGAACACTC	55	1700
AIV (H7)-R	TTAGTAGAAACAAGGGTGTTTTTTCCAAACTTATA		
AIV (H9)-F	CCAGCAAAAGCAGGGTCAAGATGAATCCAAAT	55	1,400
AIV (H9)-R	TTAGTAGAAACAAGGGTCTTTTTCTTCATCTTAG		
DTMUV-F	AGACTGCTGGTGCAATGAGAC	55	600
DTMUV-R	CGGTACCATAATCCTCCATCTCAGC		

### Virus isolation and identification

The GoAstV-positive supernatant of the tissue samples was filtered using a 0.22 μm syringe filter (Millipore, Cork, Ireland) and inoculated in 13-day-old healthy goose embryos through the chorioallantoic membrane (CAM). The embryos were incubated at 37°C and candled daily for 6 days. The embryos that died within 24 h were considered non-specific. The CAM homogenates were harvested for further analysis (Wei et al., 2020). We also performed RT-PCR to determine the presence of GoAstV using primers GoAstV-2-F and GoAstV-2-R listed in Table 1.

### Electron microscopy

Electron microscopy (EM) was performed to observe the virus based on previous studies (Wang et al., 2021). For this, the allantoic membrane tissue fluid was first centrifuged at 7,000 *g* for 30 min at 4°C, and then, ultracentrifuged at 110,000 *g* for 2 h at 4°C (Hitachi Koki Himac CP 100WX, Japan). The pellets were then resuspended in PBS (pH 7.4) and loaded over a preformed 10–60% sucrose gradient, after which the sucrose gradient was centrifuged at 110,000 *g* for 1 h at 4°C. The purified virus pellets were resuspended in sterile 1× PBS (pH 7.4) buffer and then negatively stained with 2% phosphotungstic acid. The grids were observed under a JEM-100 CX-II electron microscope after blotting and drying (JEOLLTD, Japan).

### Experimental reproduction of the disease

To assess the pathogenicity of the field isolates, goslings (*n* = 22, 1 day old), free of GoAstV-specific nucleic, were randomly divided into two groups (*n* = 11 goslings per group). The goslings in group I were infected with GoAstV at a dose of 10^5.25^ELD_50_, while those in group II were inoculated with PBS as the negative control. The clinical symptoms were monitored and recorded daily. RT-PCR detection and weighing were performed after 3, 6, 9, 12, and 15 dpi (days postinfection). Serum samples were collected after 5, 10, and 15 dpi, and were conducted to uric acid testing using uric acid enzymatic assay kit (Ruixin Biotechnology, Guangzhou). All birds were euthanized after 15 dpi. The liver, and kidney, spleen, and Brain samples were collected for histopathology and immunohistocchemistry (ICH) analysis as previous description (Sood et al., 2020).

### Genome sequencing and genetic analysis

The whole genome of the GoAstV isolates was amplified using six specific pairs of primers, which were designed based on the conserved regions of GoAstVs available in the GenBank database (Supplementary Table S2). Total RNA was extracted using the RNeasy kit (Magen, China), after which cDNA was synthesized by reverse transcription using the RT-PCR kit (TaKaRa, Dalian). The PCR products were purified, sequenced, and assembled using the DNASTAR program. Phylogenetic analysis was performed by the neighbor-joining method, using MEGA 7.0. The homology analysis heat maps and evolutionary trees were constructed using the features available on https://www.chiplot.online/. The genome sequences of GoAstV obtained in this study were deposited in the GenBank database under accession numbers ON400505 and ON382519–ON382522.

### Statistical analyses

All experiments were performed thrice independently, which yielded similar results. The variability between the trials was analyzed using SPSS 26.0 and GraphPad Prism 9. The differences between samples were determined by conducting Student’s t-tests, and all differences between groups were considered to be statistically significant at *p* < 0.05.

## Results

### Detection of goose astroviruses from clinical samples

**Figure 1:**
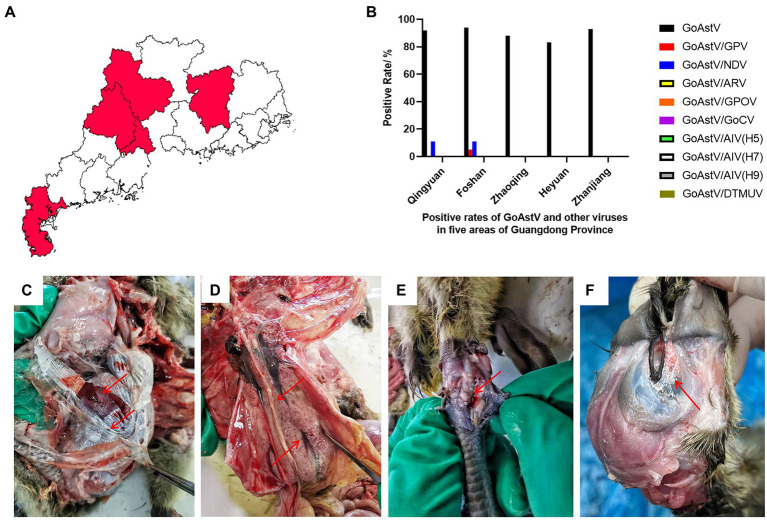
An epidemiological study was conducted with 94 clinical samples from five regions of South China. **(A,B)** Detection of GoAstV and other common viruses in five regions of South China. **(B)** The visceral surface of the abdominal cavity and the subcutaneous tissue capsule are covered by a large number of urate deposits. **(C)** Enlarged kidneys with hemorrhages and enlarged uteri with urate crystals filling the lumen. **(D)** Swollen joints with urate in the joint cavity. **(E)** The orbit is surrounded by urate deposits. **(F)** The edge of the orbit is swollen and the orbit is covered with white urate deposits.

To detect GoAstV in the waterfowl industry, 94 clinical samples were analyzed, by PCR or RT-PCR, to detect GoAstV, NDV, ARV, GPOV, GoCV, GPV, AIV (H5, H7, and H9) and DTMUV. Among them, 87 (92.6%) clinical samples tested positive for GoAstV by RT-PCR and sequencing. The positive test rates for GoAstV in Qingyuan City, Foshan City, Zhaoqing City, Heyuan City, and Zhanjiang City were 92.3% (24/26), 94.7% (18/19), 88.2% (15/17), 85.7% (12/14), and 100% (18/18), respectively (Figures 1A,B). Co-infection of GoAstV and NDV was detected in Qingyuan City and Foshan City, with positive test rates of 11.5 and 10.5%, respectively (Figure 1B). Co-infection of GoAstV and GPV was found in Foshan City, with a test positive rate of 5.3% (1/19; Figure 1B). No corresponding nucleotide fragments were observed for ARV, GPOV, GoCV, AIV (H5, H7, and H9), and DTMUV (Figure 1B).

Around 60–80% of the affected goslings exhibited depression and decreased appetite. Necropsy results showed white urate in all abdominal organs, subcutaneous tissues, and the orbit, along with blood stasis in the heart, liver, and kidneys of the goslings (Figures 1C–F).

### Virus isolation and identification

**Figure 2:**
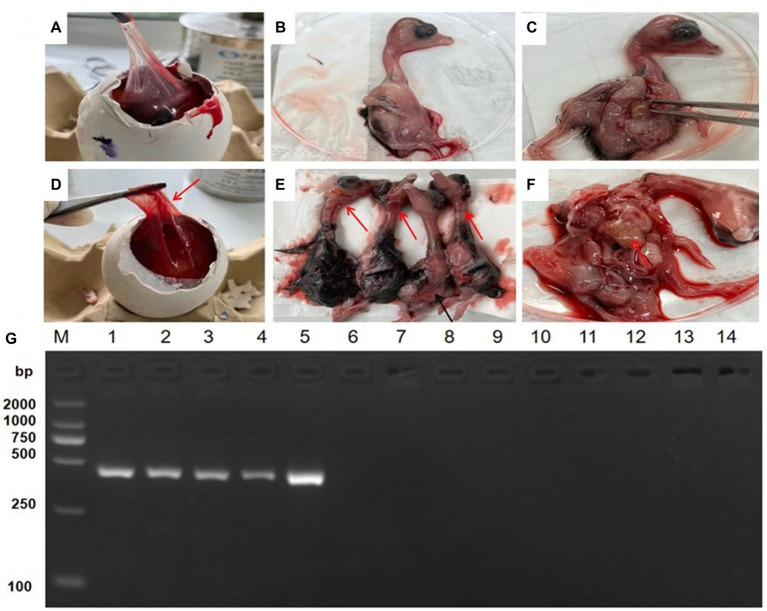
Isolation and identification of GoAstV isolates. **(A–C)** Healthy goose embryo control. **(D)** Infected embryos with a thick allantoic membrane. **(E)** Severe hemorrhages throughout the body. **(F)** Mottled necrotic foci in the infected embryo liver. **(G)** PCR identification of isolates from the allantoic membrane homogenate. M.2000 Marker; 1. GoAstV-GD2101; 2. GoAstV-GD2102; 3. GoAstV-GD2103; 4. GoAstV-GD2104; 5. GoAstV-GD2105; 6. GPV; 7. NDV; 8. ARV; 9. GPOV; 10. GoCV; 11. AIV (H5); 12. AIV (H7); 13. AIV (H9); 14. DTMUV.

**Figure 3:**
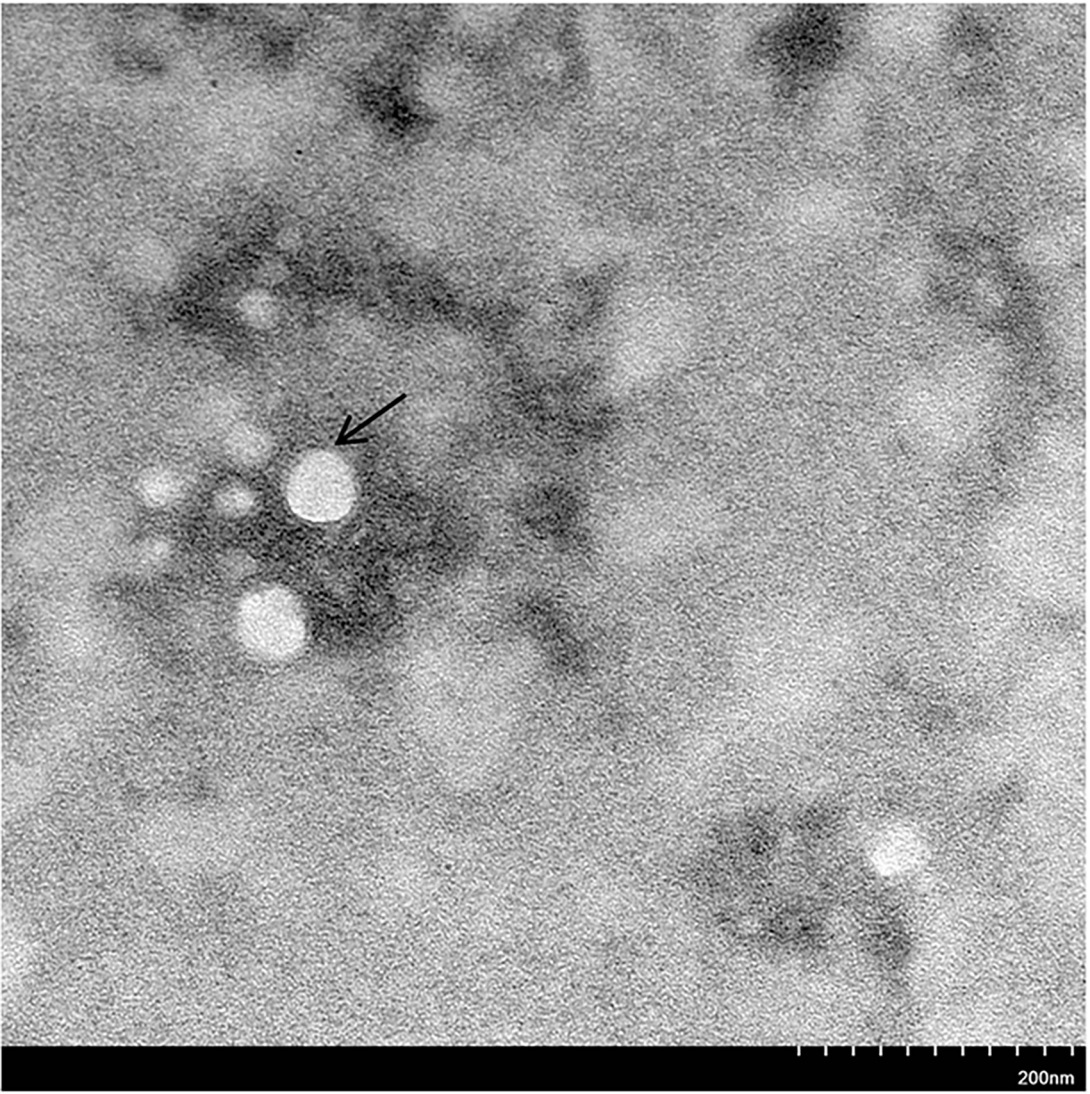
Virus particles under the electron microscope. The Black arrow indicates the novel goose astrovirus virion, which is spherical without an envelope and 23 nm in size. Original magnification: × 50,000. Bar: 200 nm.

To isolate field GoAstV from different farms, the treated supernatant was inoculated in 13-day-old healthy goose embryos. No death occurred during the initial three passages, but the fourth passage caused 40–60% mortality in the goose embryos. All dead embryos had a thick allantoic membrane, severe hemorrhages, and congestion spots (Figures 2A–F). Finally, five GoAstv isolates were successfully isolated and labeled as GoAstV-GD2101, GoAstV-GD2102, GoAstV-GD2103, GoAstV-GD2104, and GoAstV-GD2105 (Figure 2G). The purified virus particles were spherical, without a capsule, and approximately 23 nm in size, as determined by EM (Figure 3).

### Phylogenetic analysis

**Figure 4:**
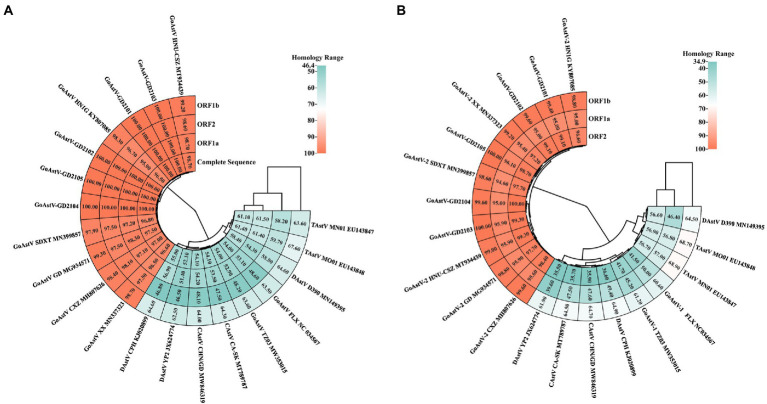
Sequence homology between five GoAstV isolates and other avian viruses. Nucleotide homology and amino acid homology among five GoAstV isolates and other species of Astroviruses were determined using the MegAlign software, and the heat map was later constructed on the website https://www.chiplot.online/. **(A)** Nucleotide homology. **(B)** Amino acid homology.

To investigate the ecology of the newly identified GoAstV strains, their whole genomes, and individual (viral protein) genes were compared to those of other AAstVs (Supplementary Table S1). The results demonstrated that the nucleotide sequence similarities among the five GoAstV isolates were 97.4–98.8%, 98.6–100%, 98.1–99.8%, and 96.7–100% for the whole genome, ORF1a, ORF1b, and ORF2, respectively, which showed that the GoAstV isolates were highly similar to each other, although they were prevalent in five different regions of Guangdong Province (Figure 4A). The five isolates showed high amino acid sequence homologies (Figure 4B), ranging from 95.7 to 99.6%, with the representative GoAstV-2 strain, while they had low homologies, ranging from 34.9 to 68.9%, with other AAstVs, which included GoAstV-1, DAstV-1, TAstV, and CAstV. These results indicated that the isolates phylogenetically belonged to the GoAstV-2 lineage.

**Figure 5:**
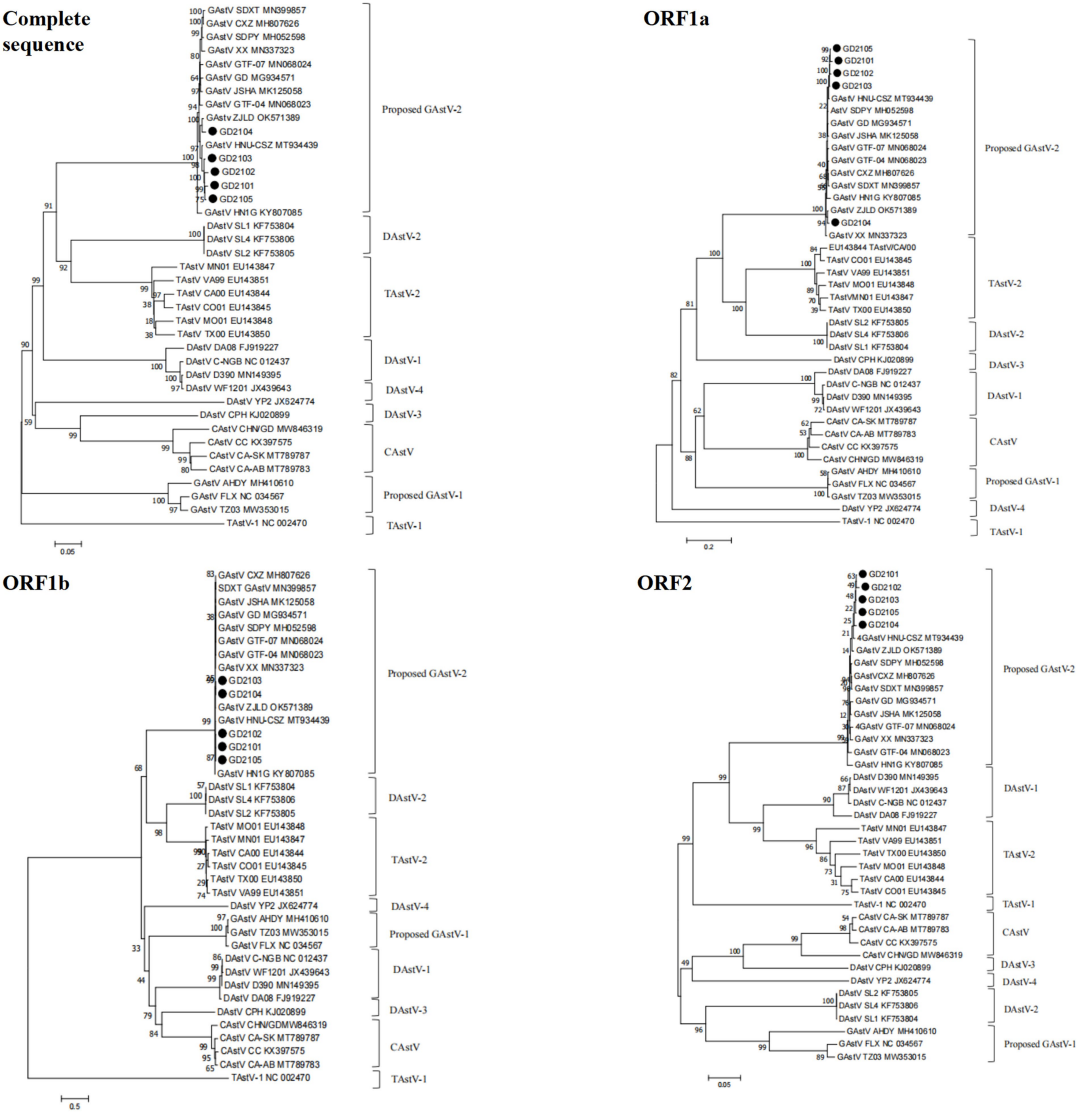
Genetic evolution analysis between five GoAstV isolates and other avian astroviruses. The evolutionary trees were generated using MEGA7.0 and the adjacency method was used with 1,000 bootstrap repeats. The black dots represent the strains isolated in this study.

To further evaluate the evolutionary relationship between the GoAstVs and other AAstVs, the whole genomes and the ORF1a, ORF1b, and ORF2 genes of emerging GoAstVs and representative strains were analyzed. The results showed that the whole genome and the ORF1a, ORF1b, and ORF2 genes of the five GoAstV isolates were clustered on a single branch, along with the recently published GoAstV-2 strains (Figure 5).

### Reproduction of gout with experimental goose astroviruses infection

**Figure 6:**
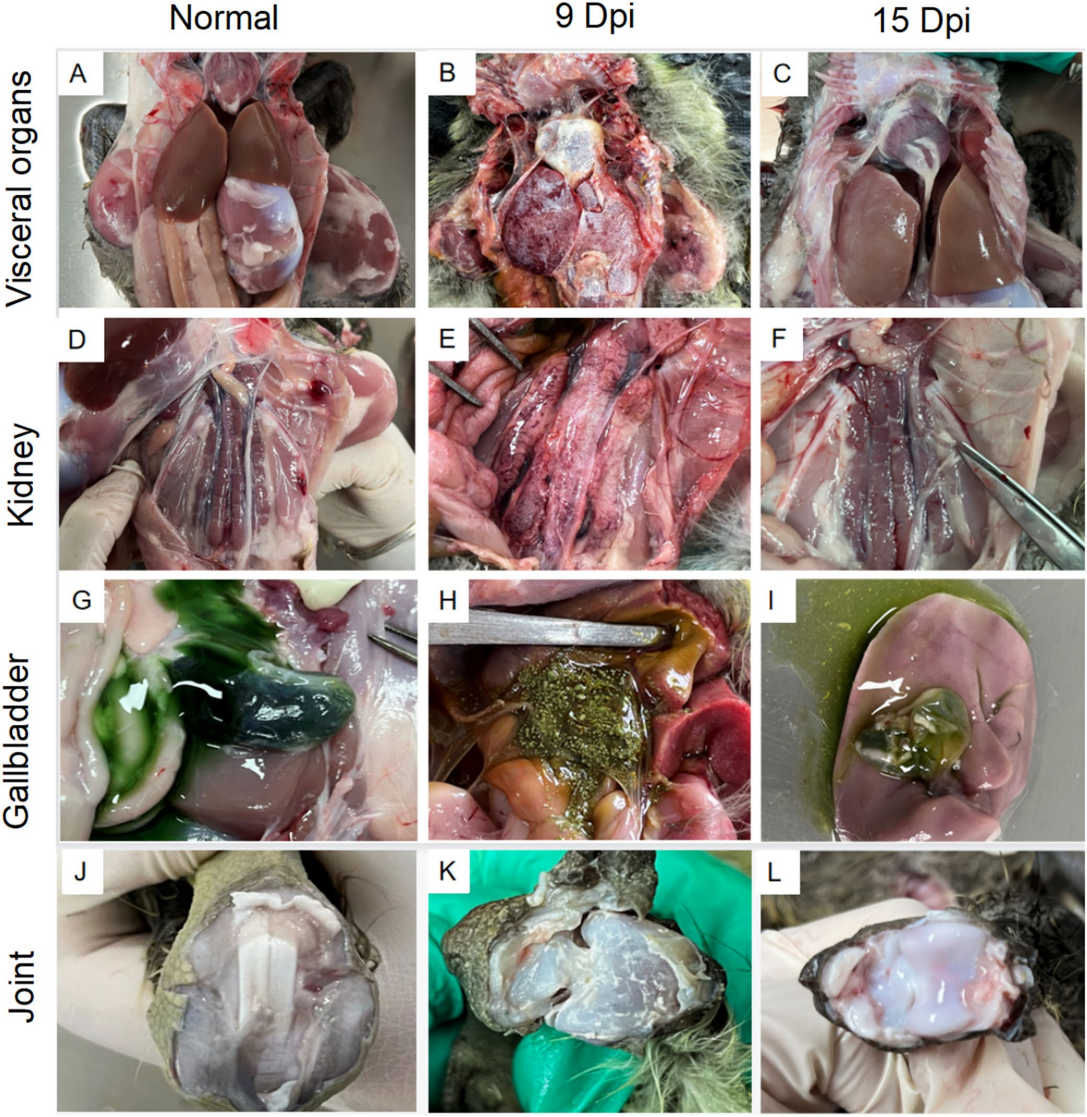
Gross lesions in lethal and non-lethal goslings infected with the GoAstV-GD2101 isolate. **(A,D,G,J)** Control group. **(B,E,H,K)** Urate deposits in the liver, kidneys, gallbladder, and joints after 9 dpi. **(C,F,I,L)** Urate deposits in the liver, kidneys, gallbladder, and joints after 15 dpi.

**Figure 7:**
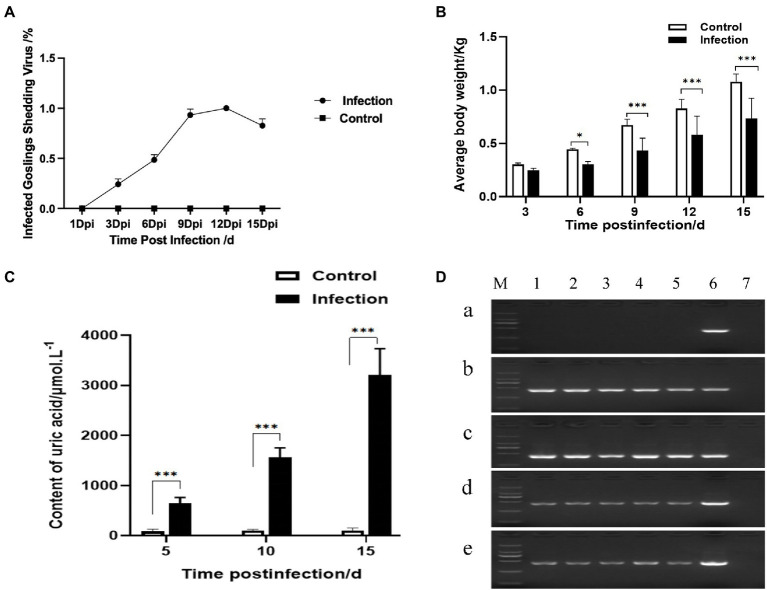
Viral shedding, growth performance, uric acid content, and viral RNA detection after GoAstV infection. **(A)** Viral shedding, **(B)** body weight monitoring, **(C)** uric acid level determination, and **(D)** GoAstV in different organs of goslings after challenge. M: 2000 DNA Marker; 1: heart; 2: liver; 3: spleen; 4: kidney; 5: brain; 6: GoAstV-positive (451 bp); 7: negative. **(A)** Healthy goslings, **(B)** 8 dpi dead goslings, **(C)** 9 dpi dead goslings, **(D)** 15 dpi alive goslings I, and **(E)** 15 dpi alive goslings II. * represents *p*<0.05 and *** represents *p*<0.005.

After being challenged with the GoAstV-GD2101 strain, the main clinical symptoms observed in infected goslings were depression, white feces, a decrease in appetite, and growth retardation after 3 dpi. The infected birds died, which peaked after 9 dpi (mortality of 36.3%). After 9 dpi and 15 dpi, the gross lesions were similar to those in naturally infected goslings, including urate deposits in the internal organs (Figures 6A-I) and hemorrhage on joints (Figures 6J-L). Virus shedding was observed after 3 dpi, which peaked after 9–12 dpi (Figure 7A). Additionally, there was a significant difference in the body weight of the birds between the infected group and the control group, which became apparent after 6 dpi (Figure 7B). The uric acid content in the serum of goslings from the infected group gradually increased and was significantly higher than that in the control group (Figure 7C). The novel GoAstV strain was re-detected from the affected goslings by RT-PCR analysis (Figure 7D).

### Histopathology and immunohistocchemistry analysis of goose astroviruses-infected goslings

**Figure 8:**
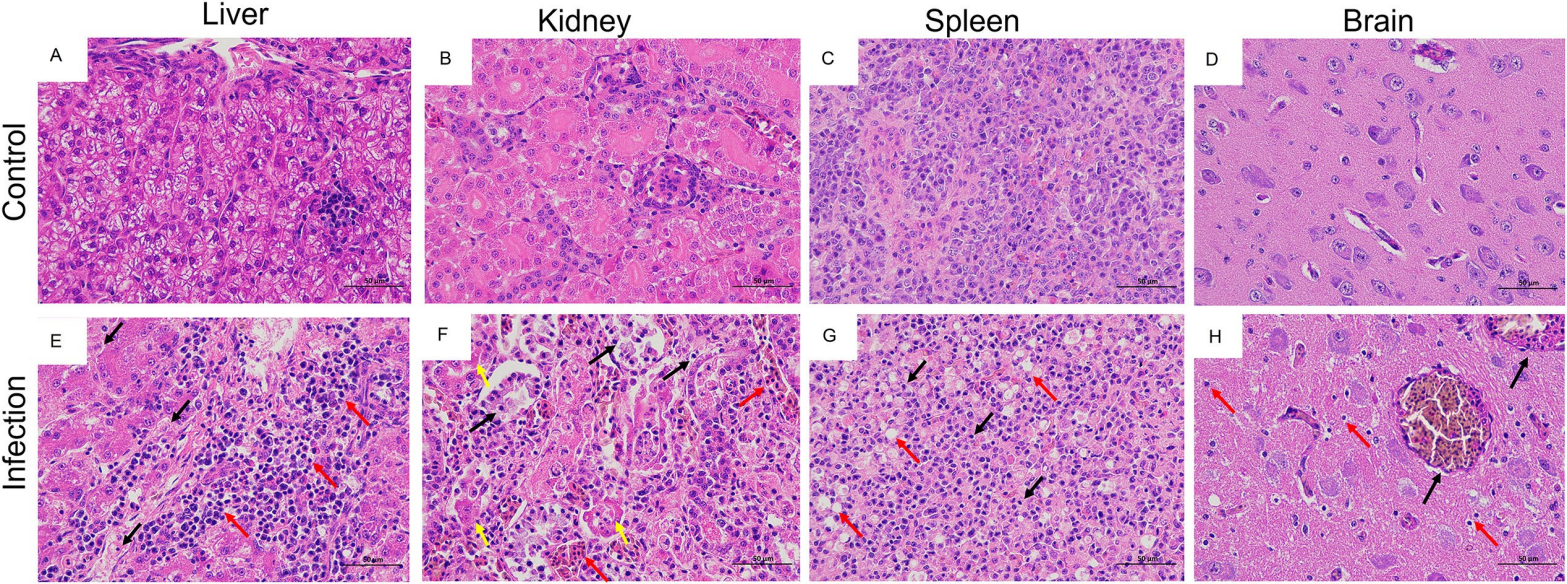
Histopathological changes in goslings, naturally infected and experimentally infected with GoAstV-GD2101. **(A–D)** Healthy liver, kidneys, spleen, and brain from the control group. **(E)** A slight loosening of hepatocyte cytoplasm (black arrow) and hepatocyte swelling (red arrow; H&E). **(F)** Severe exfoliation of renal tubular epithelial cells (black arrow), increased eosinophilia of the cytoplasm and a lumen filled with urate deposits (yellow arrow), small area of renal tubulointerstitial stasis dilatation (red arrow; H&E). **(G)** The nuclei of the spleen cells were pyknotic, deeply stained, or fragmented (black arrow), along with a large number of macrophages red arrow; H&E). **(H)** Limited congestion and dilation of blood vessels in the brain (black arrow) and diffuse proliferation of microglial cells (red arrow; H&E).

**Figure 9:**
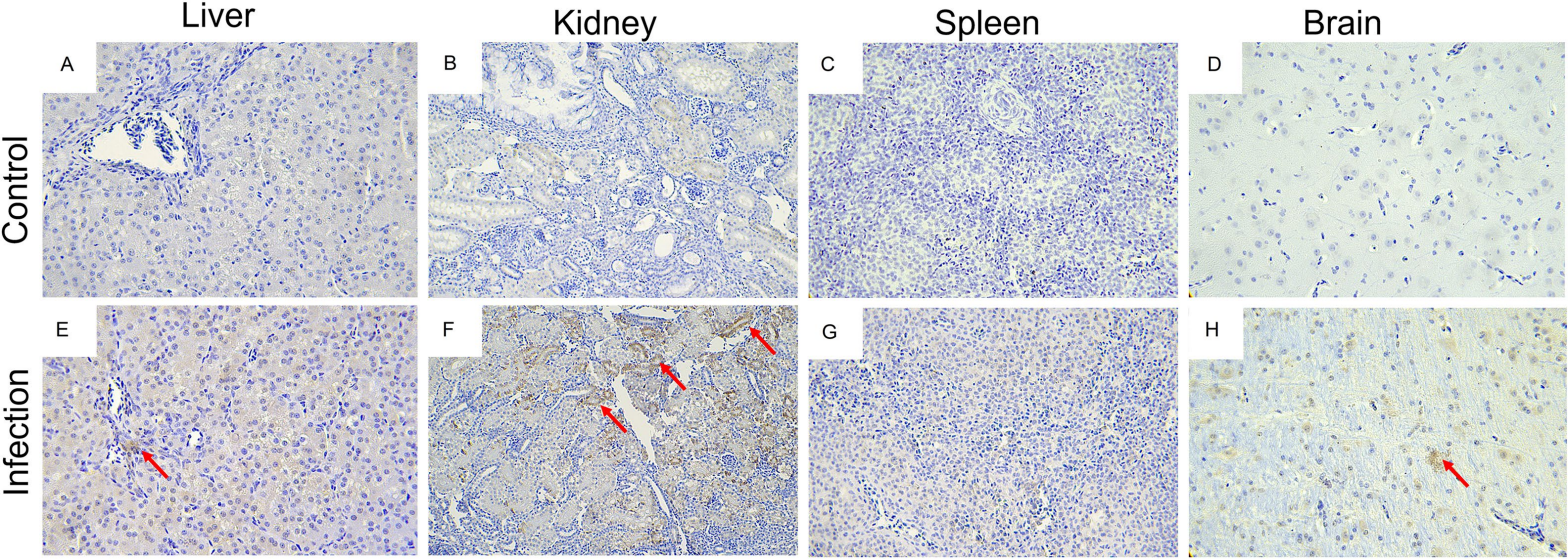
Immunohistochemistry analysis of GoAstV infected tissues. Tissue sections were deparaffinating, hydrated, water-bath heated for antigen retrieval, blocked with the addition of 3% hydrogen peroxide for immunohistochemistry, probed with indicated antibodies, and stained with indicated reagent. No positive signals were observed in the liver **(A)**, kidneys **(B)**, spleen **(C)**, and brain **(D)** of goslings in the control group. Positive signals were observed in the liver **(E)**, kidneys **(F)**, spleen **(G)**, and brain **(H)** of goslings in the challenge group.

Histopathologically, the most prominent features of this disease were observed in the liver, kidneys, spleen, and brain of infected goslings. For example, the infiltration of inflammatory cells in the liver (Figure 8E), necrosis and degeneration of renal epithelial cells in kidney (Figure 8F), diffuse hemorrhage and necrosis in the spleen (Figure 8G, and diffuse proliferation of the microglia in the brain (Figure 8H). No obvious histological lesions were found in the negative group (Figures 8A–D). ICH analysis was performed using mouse anti-GoAstV capsid protein, no positive signals were observed in the control group (Figures 9A-D), strong nuclear signal were observed in kidneys (Figure 9F), liver (Figure 9E), spleen (Figure 9G), and brain (Figure 9H) of infected goslings, which confirmed the GoAstV infection in these tissues. These findings provided strong evidence for the pathogenicity of GoAstV in goslings.

## Discussion

Since 2016, frequent outbreaks of gout have occurred in goose farms across China (Yang et al., 2018; Zhang P. et al., 2022). Due to advancements in the poultry industry, the risk factors of nutritional, secondary, and toxic gout in geese have decreased significantly. Many studies have reported that gout in geese is mainly caused by viral factors. Zhang identified and isolated GoAstV from visceral tissue samples from geese that died of gout (Zhang et al., 2018). Many studies from around the world have also shown that GoAstV is closely related to gosling gout, causing growth repression, severe visceral urate deposition, and even death, indicating that GoAstV is an important factor in gosling gout infection (An et al., 2020; Zhang F. et al., 2022). In this study, five strains of GoAstV were isolated from goose flocks with gout disease using goose embryos. The novel strains could infect embryonic development, reduce hatchability, and cause severe hemorrhages in the goose embryos. We also replicated the clinical symptoms of the natural infection with GoAstV-GD2101, confirming that the gout-causing pathogen in goslings was the goose astrovirus. Goose gout occurs due to hyperuricemia, causing renal injury due to uric acid excretion disorder or excessive uric acid production (Liu et al., 2022). When goslings are infected by GoAstV, the uric acid content in serum increases considerably, which affects transporters such as anion transporter (OAT), drug resistance-associated protein 4 (MRP4), sodium phosphate transporter (NPT1), and the Na-K-ATP pump in the renal excretory system of goslings. This, in turn, increases uric acid deposition, causing gout (Hasegawa et al., 2007; Chen et al., 2020). Therefore, drugs that protect the liver and kidneys might counter the impaired functions of these organs. In this study, uric acid deposits were found in multiple organs in the abdominal cavity of infected geese during 4–15 dpi after being challenged with GoAstV-GD2101. The uric acid levels in the goslings after 15 dpi were as high as 3,800 μmol/L, indicating that the kidney was the main target organ of GoAstV (Koci et al., 2000; Wu et al., 2020).

The results of phylogenetic analyses based on the whole genomes and ORF2 sequences of GoAstV and other reference AAstVs showed that GoAstV could be classified into two distinct clades: GoAstV-1 and GoAstV-2 (Wang et al., 2021). The gout-associated GoAstVs isolated from geese all clustered in GoAstV-2, with only a few GoAstV-1 strains, isolated from ducks, reported to result in spontaneous gout disease in ducklings (Wei et al., 2020; Chen et al., 2021). In this study, the phylogenetic trees based on the whole genome and the ORF1a and ORF1b genes showed that the isolates and the recently published GoAstV-2 strain all clustered with TAstV-2 and DAstV-2 to form sister branches. On the other hand, the phylogenetic analysis of the ORF2 gene showed that they were closely related to TAstV-2 and DAstV-1, which clustered with the TAstV-1 strain. These findings indicated that the origin of GoAstV-2 is complex, and it might be derived from different species of avian AstVs.

To summarize, we investigated the circulation of GoAstVs, which caused gout in breeder goose flocks in the Guangdong Province. Based on the molecular analysis, we identified the major virus as GoAstV-2. Our findings provided new information on the molecular epidemiology and pathogenicity of GoAstVs and might elucidate new strategies for effectively controlling the virus.

## Data availability statement

The datasets presented in this study can be found in online repositories. The names of the repository/repositories and accession number(s) can be found in the article/Supplementary material.

## Ethics statement

The animal study was reviewed and approved by the Committee of the Ethics on Animal Care and Experiments at South China Agricultural University (approval ID: SYXK-2019–0136).

## Author contributions

FC and JX conceived and designed the experiments. JX, LG, and PZ contributed to the collection of the samples. ZC, LG, PZ, and ZT performed the experiments. SC, FC, WL, LY, ZY, and MJ guided the experiments and contributed substantially to the manuscript. All authors contributed to the article and approved the submitted version.

## Funding

This study was supported by the Key Research and Development Program of Guangdong Province (No. 2019B1515210008).

## Conflict of interest

The authors declare that the research was conducted in the absence of any commercial or financial relationships that could be construed as a potential conflict of interest.

## Publisher’s note

All claims expressed in this article are solely those of the authors and do not necessarily represent those of their affiliated organizations, or those of the publisher, the editors and the reviewers. Any product that may be evaluated in this article, or claim that may be made by its manufacturer, is not guaranteed or endorsed by the publisher.
