# Supplementary material for: Isolation, identification, and pathogenicity analysis of newly emerging gosling astrovirus in South China
Source: Front Microbiol. 2023 Feb 27;14:1112245. doi: 10.3389/fmicb.2023.1112245 (PMC10008898; doi:10.3389/fmicb.2023.1112245)
Supplement: Supplementary file 2 [file Table_2.DOCX]

## Table S2. Specific primers for GoAstV-2 whole-genome sequencing.

| Primers name | Sequence (5′→3′) | Annealing temperature | Product  size (bp) |
| --- | --- | --- | --- |
| 1F | GCATGGGGAAACAGCGATATG | 58℃ | 1207 |
| 1R | TGGGCAACTTAAACCACCAT |  |  |
| 2F | GGTTGAGAAGCTCATACCGC | 58℃ | 1743 |
| 2R | CTGACACGGATGAGGTGTAA |  |  |
| 3F | ATGAAGAAGGTGCGGAAGAG | 58℃ | 1346 |
| 3R | TATATACTGGATCAGCACACAAG |  |  |
| 4F | CACTTTGCCCGACTATATAGA | 58℃ | 1326 |
| 4R | GCATTTTCGCACAAGATCCT |  |  |
| 5F | GTCTCTGATGATATTGAGGGTATG | 58℃ | 1562 |
| 5R | TCCCGTGAAAGGACAGTGGTA |  |  |
| 6F | TCCACAGAAGTGGTCGAGAC | 58℃ | 1355 |
| 6R | TGCTTAAAAATCACATTTGATTC |  |  |
